# Neurobehavioral Effects of Levetiracetam in Patients with Traumatic Brain Injury

**DOI:** 10.3389/fneur.2013.00195

**Published:** 2013-12-02

**Authors:** Jared F. Benge, Richard A. Phenis, Abigail Bernett, Daniel Cruz-Laureano, Batool F. Kirmani

**Affiliations:** ^1^Department of Neurology, Scott & White Healthcare, Texas A&M Health Science Center College of Medicine, Temple, TX, USA

**Keywords:** neurobehavioral effects, levetiracetam, traumatic brain injury, neurological functioning, neurobehavioral outcomes

## Abstract

Moderate to severe traumatic brain injury (TBI) is one of the leading causes of acquired epilepsy. Prophylaxis for seizures is the standard of care for individuals with moderate to severe injuries at risk for developing seizures, though relatively limited comparative data is available to guide clinicians in their choice of agents. There have however been experimental studies which demonstrate potential neuroprotective qualities of levetiracetam after TBI, and in turn there is hope that eventually such agents may improve neurobehavioral outcomes post-TBI. This mini-review summarizes the available studies and suggests areas for future studies.

## Introduction

Traumatic brain injury (TBI) refers to a spectrum of neurological injury resulting from external forces applied to the brain that cause changes in neurological functioning ranging from a brief alteration of consciousness (known as a mild TBI or concussion) to severe TBI which is marked by extended periods of coma or altered consciousness (1). The severity of the initial injury is a fairly reliable predictor of neurobehavioral outcomes, with more severe injuries likely leading to permanent neurobehavioral impairments (2). For individuals who survive the acute phase of a moderate to severe TBI, a period of functional recovery occurs for up to 2 years post-injury (3). Despite the potential for recovery, those who survive moderate to severe TBI frequently experience a host of persistent neurobehavioral symptoms, such as cognitive deficits, difficulties with social judgment, fatigue, and mood changes (4).

In addition to the permanent neurobehavioral problems that can result from moderate to severe TBI, 5–20% of patients who sustain a severe TBI will develop seizures, a phenomenon known as post-traumatic epilepsy (PTE) (5, 6). Injury characteristics impact risk for PTE, with 50% of patients sustaining penetrating head injuries developing seizures, while closed head injuries appear less likely to develop such complications (7). The presence of visible contusions, hemorrhages, longer coma duration, and older age all also increase the risk for PTE (8).

Post-traumatic epilepsy may account for up to 5% of all cases of epilepsy in general (6). It is unclear which if any neurophysiological markers are most strongly associated with outcomes, as well as the development of PTE (9, 10), but there is increasing evidence that PTE is associated with worse functional outcomes in general (11). Experimental models have revealed that the post-injury time period is marked by a host of excitatory neurochemical changes as well as structural brain changes such as cellular loss and changes in organization which may foster the development of PTE (12). Therefore, the acute stage presents an opportunity to intervene prophylactically with anti-epileptic medications in hopes of preventing or limiting seizure activity. Although there remains debate about whether prevention of seizures in the acute post-TBI time period actually prevents the development of epilepsy over longer time periods (13), current guidelines recommend anti-epileptic drug (AED) use in at risk patients during the first 7 days post-injury to prevent acute seizures (14).

The choice of AED agent utilized in severe TBI cases has begun to shift over the years. Because of its availability in intravenous format and clinical utility, phenytoin (PHT) was historically utilized for PTE prophylaxis, despite its need for ongoing clinical monitoring and potential for serious adverse side effects (15). However, since levetiracetam (LEV) became available in an intravenous formulation, it has been increasingly utilized because it requires no loading dose or ongoing monitoring (16). Recent meta-analysis (17) and clinical data (18) suggest that both agents are equally effective in preventing post-traumatic seizures during the first 7 days post-injury, though to date no data is available to indicate the agents ability to prevent PTE.

Although there are encouraging findings for LEV’s use to prevent acute seizures following TBI, the agents impact on neurobehavioral outcomes has been relatively unexplored. As noted above, moderate to severe TBI by itself is associated with a host of neurobehavioral symptoms which vary in severity from patient to patient and evolve over the course of recovery. Memory impairments, difficulties with executive functioning and social regulation, fatigue, depression, and irritability/aggression are common post-TBI sequelae (4). Given that some of these symptoms have been associated with AED use in general (19, 20), it is important to fully understand any potential interactive or additive effects in the TBI population in an attempt to avoid or mitigate any untoward clinical outcomes.

## Neurobehavioral Impacts of LEV in Populations Other than TBI

Levetiracetam has proven to be a popular agent in many neurological populations, in part because it has been relatively well-tolerated from a neurobehavioral standpoint. However, a review of the evidence from epilepsy samples suggests that LEV treatment is associated with changes in emotional functioning. Specifically, studies are suggestive of increased aggression and possibly suicidality, especially in individuals with premorbid depression or behavior problems (21–23). In children, there is some evidence that LEV use may be particularly associated with untoward behavioral outcomes. Schiemann-Delgado studied LEV in children with partial onset seizures and found that LEV was associated with stable cognitive performance versus placebo, but also mild neurobehavioral adverse effects, including increased aggression and irritability (24).

Summarizing the available data, Mbizvo and colleagues suggested that in patients with epilepsy, LEV add on treatment was associated with increased somnolence, changes in behavior in 23% of children studied (but few adults), and no significant impact on cognition (25).

From a neuropsychological standpoint, the medication seems well-tolerated. In a small (16 subject) but well-designed experiment involving healthy controls, LEV had cognitive and electrophysiological effects comparable to that of placebo, suggesting that at least over the short run in healthy subjects, it had little adverse neurobehavioral impact (26). LEV may even provide some cognitive benefit in select populations. For example, LEV has been associated with improved memory in patients with high grade gliomas (27, 28). Similarly, in a retrospective review of patients with a history of intracranial hemorrhage, patients treated with LEV were discharged home more often, had higher Glasgow coma scale (GCS) scores, and demonstrated a trend toward better global cognitive status (defined as oriented and cooperative versus not) (29).

## The Interaction of LEV and TBI on Neurobehavioral Outcomes

In contrast to other populations such as epilepsy or general neurosurgery patients, the study of the neurobehavioral profile of LEV in TBI is still in its infancy, with most data culled from recent efficacy studies. These studies tend to utilize relatively broad self or caregiver reports of neurobehavioral changes with little formal cognitive testing or more granular assessments of neurobehavioral outcomes.

What information is available, based largely on a series of papers from the same study suggests an increase in fatigue with LEV use in TBI during the acute phase. Klein et al published data on the pharmacokinetics of a PHT + LEV treatment arm and noted that around 3% of subjects discontinued the LEV secondary to somnolence (30). In a follow-up safety study, Klein and colleagues (31) found approximately 15% of their sample reported fatigue, somnolence, and headache, with most of these symptoms reported as mild in nature.

Pearl and colleagues (32) recently published data specifically evaluating the pediatric subjects from the aforementioned study, followed over 2 months and later 2 years. This study included measures of problematic behavior and depression, and interestingly there was no difference between LEV treated patients and controls on these measures. However, during active treatment, LEV patients showed higher rates of headache, fatigue, drowsiness, and irritability. Eighty-five percent of patients complained of fatigue, but only 5% rated it as severe. One patient had a psychosis which resolved with LEV discontinuation. While fatigue may be considered a minor side effect, it may interfere with participation in brain injury rehabilitation, which in turn could lead to other untoward outcomes in a TBI population, and this warrants further investigation. For example, Nair and Kadies (33) published a case study of an older individual participating in rehabilitation for a TBI who was having persistent sleep wake cycle disorder and agitation. While these symptoms had been attributed to his TBI, after removing LEV he gradually resumed a normal sleep wake cycle and had less agitation which in turn led to better participation in rehabilitation.

At a more global level, there is some evidence for better neurobehavioral outcomes in both the short and long term with LEV versus PHT. Szarflarski et al (34) studied a group of 52 patients the majority of which suffered a severe TBI in a randomized single-blinded study comparing PHT and LEV. They included global outcome measures including the disability rating scale (DRS) and Glasgow outcome scale (GOS), which assess in a broad way neurobehavioral status. In this study, there was no difference in seizure outcomes over both short term and long term outcome, and similar rates of mortality in each group. Side effect profiles were similar between groups, with LEV patients having fewer instances of a decrease in neurological status and fewer gastrointestinal problems. Most notably, the LEV patients demonstrated a statistically significant lower (better) score on the DRS and a higher (better) GOS score than their PHT matched controls. In contrast to these findings though, Jones and colleagues (35) found similar 3 and 6 months GOS outcomes when comparing PHT and LEV, and noted that their LEV patients had stronger tendencies to seizure activity on EEG (but no greater increase in seizures). Thus the potential for an actual neurobehavioral benefit to LEV use in post-TBI care remains to be definitively established.

Unfortunately at the time of this writing no studies were found which specifically evaluated the neurobehavioral impact of LEV in the chronic phase of the recovery or in individuals who had developed PTE.

## Potential for Novel Therapeutic Uses

While current work has focused on LEV as a prophylactic agent for PTE, there is a history of laboratory work as well as clinical observations suggesting LEV is a neuroprotective agent which may improve behavioral outcomes even in the absence of seizure activity. As noted above, LEV use was associated with better cognitive outcomes in brain tumor patients (27), and one study revealed LEV to be associated with improved global outcome (including neurobehavioral functioning) in severe TBI cases (34). Similarly, in the suspected prodromal phase of Alzheimer’s disease (amnestic mild cognitive impairment) LEV use was associated with reduction in hippocampal activity and paradoxically, improved cognition (36). The authors suggest that increased hippocampal activation may be a sign of potentially damaging overactivation of the brain, and that LEV may reduce this and thus preserve neurons.

Consistent with this finding, in a rat model of TBI involving controlled cortical impacts, animals treated early with LEV versus a saline control had improved motor function, increased exploratory behavior, better preserved hippocampal cells, and reduced total volume of contusions (37). The authors propose that despite LEV still not having a fully elucidated mechanism of action for the prevention of seizures, its ability to upregulate glutamate transporters may lead to increased neuroprotection as well as improved anti-epileptic impact. A similar study conducted by Wang and colleagues (38) demonstrated a similar pattern of neuroprotective effects that were not present in animals treated with fosphenytoin. If replicated and extended to humans, such a finding would support the use of LEV not just to prevent seizures but also to prevent the secondary damage of excitotoxicity in the peri-injury period.

## Directions for Future Research

Studying neurobehavioral phenomena in TBI is a complex endeavor, given the heterogeneity of initial injury, different recovery courses, and the difficulty of measuring complex phenomena such as mood, cognition, and behavior. Partialing out the impact of a medication such as LEV from the disorder itself which can result in many of the same symptoms will require careful study design. A well-designed study to evaluate the neurobehavioral impacts of LEV would have to include control and treatment groups which are carefully randomized or matched to control for the impact of variability in initial injury severity, time since injury, and relevant demographic and other medical factors (for example controlling for the presence of other neurobehaviorally active drugs such as anti-depressants and pain medications). Given the difficulty of relying on self-report in patients with potential impairments in cognition and self-awareness, multi-modal assessment end points, including neuropsychological testing and informant ratings will be necessary to adequately capture the phenomena of interest. Electrophysiological markers may be helpful to quantify the nature and extent of physiological impact of LEV in this population, and imaging techniques to quantify the interaction of specific structural abnormalities and medication effects would also be intriguing.

While much research remains to be done on establishing the efficacy or superiority of LEV for seizure prophylaxis post severe TBI, future studies may also want to move toward studying LEV as an adjunctive neuroprotective agent. Adding a longitudinal neurobehavioral component to an acute LEV vs. placebo or active control study with more granular neurobehavioral ratings for each stage of recovery (i.e., time to follow commands in the acute phase, ranging to neuropsychological evaluations later in the recovery course) would allow for evaluating LEV as a potential neuroprotective agent. Naturalistic studies which look at the subset of a sample who continue LEV treatment beyond the current 7 days window may also yield insights into a potential benefit from this medication. Finally, functional neuroimaging may provide insight into how LEV alters the functional activation and connectivity of the recovering brain, unlocking the mechanisms into its neurobehavioral impact.

## Conflict of Interest Statement

The authors declare that the research was conducted in the absence of any commercial or financial relationships that could be construed as a potential conflict of interest.

## References

[1] Faul M Xu L Wald MM Coronado VG Traumatic Brain Injury in the United States: Emergency Department Visits, Hospitalizations, and Deaths. Atlanta, GA: Centers for Disease Control and Prevention, National Center for Injury Prevention and Control (2010).

[2] Rohling ML Meyers JE Millis SR Neuropsychological impairment following traumatic brain injury: a dose-response analysis. Clin Neuropsychol (2003) 17:289–302 10.1076/clin.17.3.289.18086 14704881

[3] Dikmen S Machamer J Temkin N McLean A Neuropsychological recovery in patients with moderate to severe head injury: 2 year follow-up. J Clin Exp Neuropsychol (1989) 12:507–19 10.1080/01688639008400997 2211973

[4] Roebuck-Spencer T Sherer M Moderate and severe traumatic brain injury. In: Morgan J Richker H editors. Textbook of Clinical Neuropsychology. New York, NY: Taylor and Francis (2008). p. 411–29

[5] Temkin NR Preventing and treating posttraumatic seizures: the human experience. Epilepsia (2009) 50:10–3 10.1111/j.1528-1167.2008.02005.x 19187289

[6] Diaz-Arrastia R Agostini M Madden CJ Van Ness PC Post-traumatic epilepsy: the endophenotypes of a human model of epileptogenesis. Epilepsia (2009) 50:14–20 10.1111/j.1528-1167.2008.02006.x 19187290

[7] Pagni CA Zenga F Prevention and treatment of post-traumatic epilepsy. Expert Rev Neurother (2006) 6:1223–33 10.1586/14737175.6.8.1223 16893349

[8] Chen JW Ruff RL Eavey R Wasterlain CG Posttraumatic epilepsy and treatment. J Rehabil Res Dev (2009) 46:685–96 10.1682/JRRD.2008.09.0130 20104398

[9] Vespa PM Nuwer MR Nenov V Ronne-Engstrom E Hovda DA Bergsneider M Increased incidence and impact of nonconvulsive and convulsive seizures after traumatic brain injury as detected by continuous electroencephalographic monitoring. J Neurosurg (1999) 91:750–60 10.3171/jns.1999.91.5.0750 10541231 PMC4347935

[10] Steinbaugh LA Lindsell CJ Shutter LA Szaflarski JP Initial EEG predicts outcomes in a trial of levetiracetam vs. fosphenytoin for seizure prediction. Epilepsy Behav (2012) 23:280–4 10.1016/j.yebeh.2011.12.005 22342434

[11] Bushnik T Englander J Wright J Kolakowsky-Hayner S Traumatic brain injury with and without late posttraumatic seizures: what are the impacts in the post-acute phase: a NIDRR Traumatic Brain Injury Model Systems study. J Head Trauma Rehabil (2012) 27:E36–44 10.1097/HTR.0b013e318273375c 23131969

[12] Hunt RF Boychuk JA Smith BN Neural circuit mechanisms of post-traumatic epilepsy. Front Cell Neurosci (2013) 7:89 10.3389/fncel.2013.00089 23785313 PMC3684786

[13] Teasell R Bayona N Lippert C Villamere J Hellings C Post-traumatic seizure disorder following acquired brain injury. Brain Inj (2007) 21:201–14 10.1080/02699050701201854 17364531

[14] Chang BS Lowenstein DH Practice parameter: antiepileptic drug prophylaxis in severe traumatic brain injury: report of the Quality Standards Subcommittee of the American Academy of Neurology. Neurology (2003) 60:10–6 10.1212/01.WNL.0000031432.05543.14 12525711

[15] Winter ME Phenytoin and fosphenytoin. In: Murphy JE editor. Clinical Pharmacokinetics. Bethesda, MD: American Society of Health-System Pharmacists (2008). p. 247–64

[16] Kruer RM Harris LH Goodwin H Kornbluth J Thomas KP Slater LA Changing trends in the use of seizure prophylaxis after traumatic brain injury: a shift from phenytoin to levetiracetam. J Crit Care (2013) 28:883 10.1016/j.jcrc.2012.11.020 23566730

[17] Zafar SN Khan AA Ghauri AA Shamim M Phenytoin versus leviteracetam for seizure prophylaxis after brain injury – a meta analysis. BMC Neurol (2012) 12:30 10.1186/1471-2377-12-30 22642837 PMC3406949

[18] Inaba K Menaker J Branco BC Gooch J Okoye OT Herrold J A prospective multicenter comparison of levetiracetam versus phenytoin for early posttraumatic seizure prophylaxis. J Trauma Acute Care Surg (2013) 74:766–71 10.1097/TA.0b013e3182826e84 23425733

[19] Loring DW Marino S Meador KJ Neuropsychological and behavioral effects of anti-epilepsy drugs. Neuropsychol Rev (2007) 17:413–25 10.1007/s11065-007-9043-9 17943448

[20] Schmitz B Effects of anti-epileptic drugs on mood and behavior. Epilepsia (2006) 47:28–33 10.1111/j.1528-1167.2006.00684.x 17105456

[21] French J Edrich P Cramer JA A systematic review of the safety profile of levetiracetam: a new anti-epileptic drug. Epilepsy Res (2001) 47:77–90 10.1016/S0920-1211(01)00296-0 11673023

[22] Dinkelacker V Diet LT Widman G Lengler U Elger CE Aggressive behavior of epilepsy patients in the course of levetiracetam add-on therapy: report of 33 mild to severe cases. Epilepsy Behav (2003) 4:537–47 10.1016/j.yebeh.2003.07.008 14527496

[23] Mula M Bell GS Sander JW Suicidality in epilepsy and possible effects of antiepileptic drugs. Curr Neurol Neurosci Rep (2010) 10:327–32 10.1007/s11910-010-0117-3 20473787

[24] Schiemann-Delgado J Yang H de la Loge C Stalvey T Jones J LeGoff D A long-term open-label extension study assessing cognition and behavior, tolerability, safety, and efficacy of adjunctive levetiracetam in children aged 4 to 16 years with partial-onset seizures. J Child Neurol (2011) 27:80–9 10.1177/0883073811417183 21876066

[25] Mbizvo GK Dixon P Hutton JL Marson AG Levetiracetam add-on for drug-resistant focal epilepsy: an updated cochrane review. Cochrane Database Syst Rev (2012) 9:CD001901 10.1002/14651858.CD001901 22972056 PMC7061650

[26] Meador KJ Gevins A Leese P Otoul C Loring DW Neurocognitive effects of brivaracetam, levetiracetam, and lorazepam. Epilepsia (2011) 52:264–72 10.1111/j.1528-1167.2010.02746.x 20887370

[27] de Groot M Douw L Sizoo EM Bosma I Froklage FE Heimans JJ Leveitiracetam improves verbal memory in high-grade glioma patients. Neuro Oncol (2013) 15:216–23 10.1093/neuonc/nos288 23233537 PMC3548582

[28] Bernett A Phenis R Fonkem E Aceves J Kirmani B Cruz-Laureano D Neurobehavioral effects of levetiracetam in brain tumor related epilepsy. Front Neurol (2013) 4:99 10.3389/fneur.2013.00099 23885253 PMC3717617

[29] Taylor S Heinrichs RJ Janzen JM Ehtisham A Levetiracetam is associated with improved cognitive outcome for patients with intracranial hemorrhage. Neurocrit Care (2011) 15:80–4 10.1007/s12028-010-9341-6 20890680

[30] Klein P Herr D Pearl PL Natale J Levine Z Nogay C Results of phase 2 safety and feasibility study of treatment with levetiracetam for prevention of posttraumatic epilepsy. Arch Neurol (2012) 69:1290–5 10.1001/archneurol.2012.445 22777131 PMC4798941

[31] Klein P Herr D Pearl PL Natale J Levine Z Nogay C Results of phase II pharmacokinetic study of levetiracetam for prevention of post-traumatic epilepsy. Epilepsy Behav (2012) 24:457–61 10.1016/j.yebeh.2012.05.011 22771222 PMC4561854

[32] Pearl PL McCarter R McGavin CL Yu Y Sandoval F Trzcinski S Results of a phase II levetiracetam trial following acute head injury in children at risk for posttraumatic epilepsy. Epilepsia (2013) 54:e135–7 10.1111/epi.12326 23876024 PMC3769484

[33] Nair CV Kadies MA Case report: sleep wake cycle disorder and agitation associated with levetiracetam in an elderly patient with traumatic brain injury. Br J Med Pract (2012) 5:3

[34] Szaflarski JP Sangha KS Lindsell CJ Shutter LA Prospective, randomized, single-blinded comparative trial of intravenous levetiracetam versus phenytoin for seizure prophylaxis. Neurocrit Care (2010) 12:165–72 10.1007/s12028-009-9304-y 19898966

[35] Jones KE Puccio AM Harshman KJ Falcione B Benedict N Jankowitz BT Levetiracetam versus phenytoin for seizure prophylaxis in severe traumatic brain injury. Neurosurg Focus (2008) 25:E3 10.3171/FOC.2008.25.10.E3 18828701 PMC3705765

[36] Bakker A Krauss GL Albert MS Speck CL Jones LR Stark CE Reduction of hippocampal hyperactivity improves cognition in amnestic mild cognitive impairment. Neuron (2012) 74:467–74 10.1016/j.neuron.2012.03.023 22578498 PMC3351697

[37] Zou H Brayer SW Hurwitz M Niyonkuru C Fowler LE Wagner AK Neuroprotective, neuroplastic, and neurobehavioral effects of daily treatment with levetiracetam in experimental traumatic brain injury. Neurorehabil Neural Repair (2013) 27(9):878–88 10.1177/1545968313491007 23812605

[38] Wang H Gao J Lassiter TF McDonagh DL Sheng H Warner DS Levetiracetam is neuroprotective in murine models of closed head injury and subarachnoid hemorrhage. Neurocrit Care (2006) 5:71–8 10.1385/NCC:5:1:71 16960300

